# Crystal structure of (*E*)-3-[4-(benzyl­idene­amino)-5-sulfanyl­idene-3-(*p*-tol­yl)-4,5-di­hydro-1*H*-1,2,4-triazol-1-yl]-3-(4-meth­oxy­phen­yl)-1-phenyl­propan-1-one

**DOI:** 10.1107/S2056989015023804

**Published:** 2015-12-24

**Authors:** Hewen Wang

**Affiliations:** a College of Chemical Engineering, Huanggang Normal University, Huanggang 438000, People’s Republic of China

**Keywords:** crystal structure, 1,2,4-triazole derivative, Mannich reaction

## Abstract

The title compound, C_32_H_28_N_4_O_2_S, crystallizes as a racemate. In the mol­ecule, the bond-angle sum at the C atom of the sulfanyl­idene entity bound to the triazole ring is 360°, with an annular N—C—N bond angle of 102.6 (2)° and two larger N—C—S angles of 127.3 (2) and 130.1 (2)°. The essentially planar 1,2,4-triazole ring (r.m.s. deviation = 0.013 Å) is nearly perpendicular to the phenylpropanone and methoxyphenyl rings , making dihedral angles of 76.9 (2) and 85.2 (2)°, respectively and subtends dihedral angles of 17.6 (2) and 40.3 (2)° with the tolyl and benzylideneamino rings, respectively. There is no π–π stacking between the mol­ecules. The crystal packing is dominated by weak C—H⋯O and C—H⋯N inter­actions, leading to a three-dimensional network structure. An intra­molecular C—H⋯S inter­action also occurs.

## Related literature   

Mannich base derivatives are used in numerous applications, *e.g.* in agrochemistry, pharmacy and polymer chemistry. Synthesis and crystal structures of such compounds were reported recently (Wang *et al.*, 2011 ▸; Shams *et al.*, 2011 ▸). Bond lengths and angles of the title compound are comparable with related 1,2,4-triazole-5(4*H*)-thione derivatives (Al-Tamimi *et al.*, 2010 ▸; Fun *et al.*, 2009 ▸; Tan *et al.*, 2010 ▸). For the crystal structures of other triazole derivatives, see: Zhao *et al.* (2010 ▸); Gao *et al.* (2011 ▸).
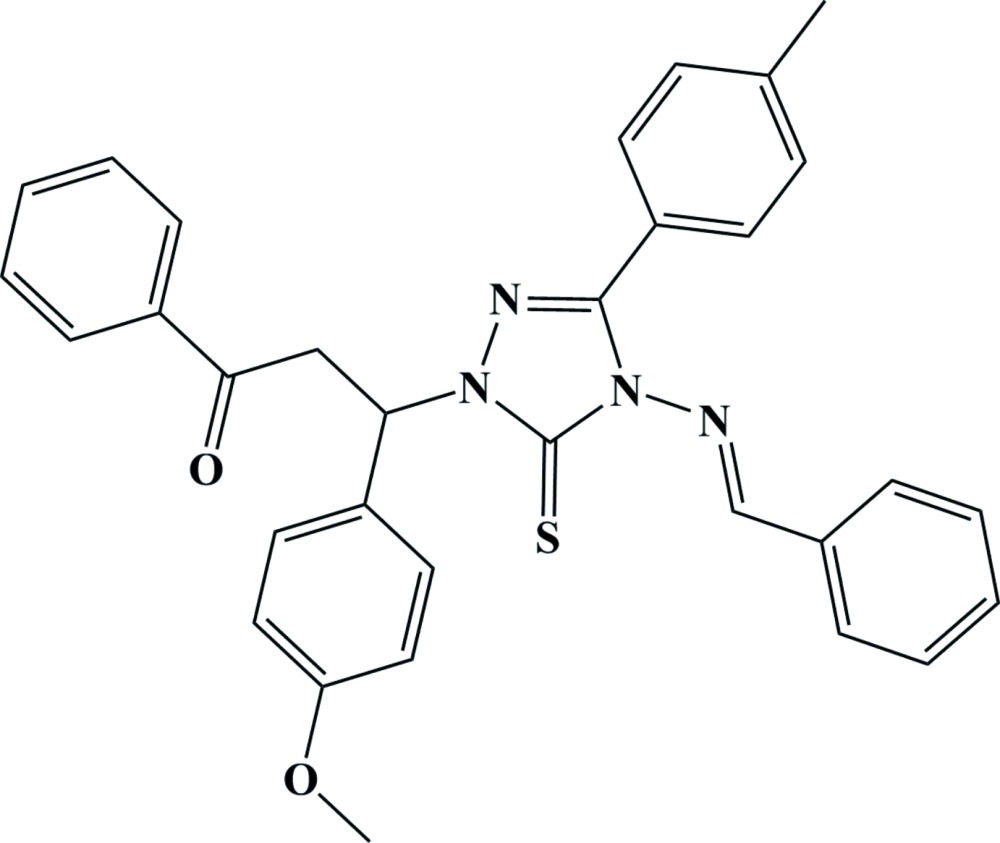


## Experimental   

### Crystal data  

C_32_H_28_N_4_O_2_S
*M* *_r_* = 532.64
Triclinic, 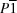

*a* = 6.2343 (6) Å
*b* = 14.6968 (14) Å
*c* = 15.0540 (17) Å
α = 90.234 (9)°
β = 92.612 (13)°
γ = 99.497 (10)°
*V* = 1358.9 (2) Å^3^
*Z* = 2
Mo *K*α radiation
μ = 0.16 mm^−1^
*T* = 113 K
0.20 × 0.18 × 0.12 mm

### Data collection  

Rigaku Saturn CCD area-detector diffractometer
Absorption correction: multi-scan (*CrystalClear*; Rigaku/MSC, 2005 ▸) *T*_min_ = 0.969, *T*_max_ = 0.982
11718 measured reflections
4778 independent reflections
3450 reflections with *I* > 2σ(*I*)
*R*_int_ = 0.057

### Refinement  

*R*[*F* ^2^ > 2σ(*F* ^2^)] = 0.065
*wR*(*F* ^2^) = 0.126
*S* = 1.08
4778 reflections
354 parameters
H-atom parameters constrained
Δρ_max_ = 0.23 e Å^−3^
Δρ_min_ = −0.25 e Å^−3^

### 

Data collection: *CrystalClear* (Rigaku/MSC, 2005 ▸); cell refinement: *CrystalClear*; data reduction: *CrystalClear*; program(s) used to solve structure: *SHELXS97* (Sheldrick, 2008 ▸); program(s) used to refine structure: *SHELXL97* (Sheldrick, 2008 ▸); molecular graphics: *SHELXTL* (Sheldrick, 2008 ▸); software used to prepare material for publication: *CrystalStructure* (Rigaku/MSC, 2005 ▸).

## Supplementary Material

Crystal structure: contains datablock(s) global, I. DOI: 10.1107/S2056989015023804/wm5250sup1.cif

Structure factors: contains datablock(s) I. DOI: 10.1107/S2056989015023804/wm5250Isup2.hkl

Supporting information file. DOI: 10.1107/S2056989015023804/wm5250Isup3.cml

**Fig. 1.**
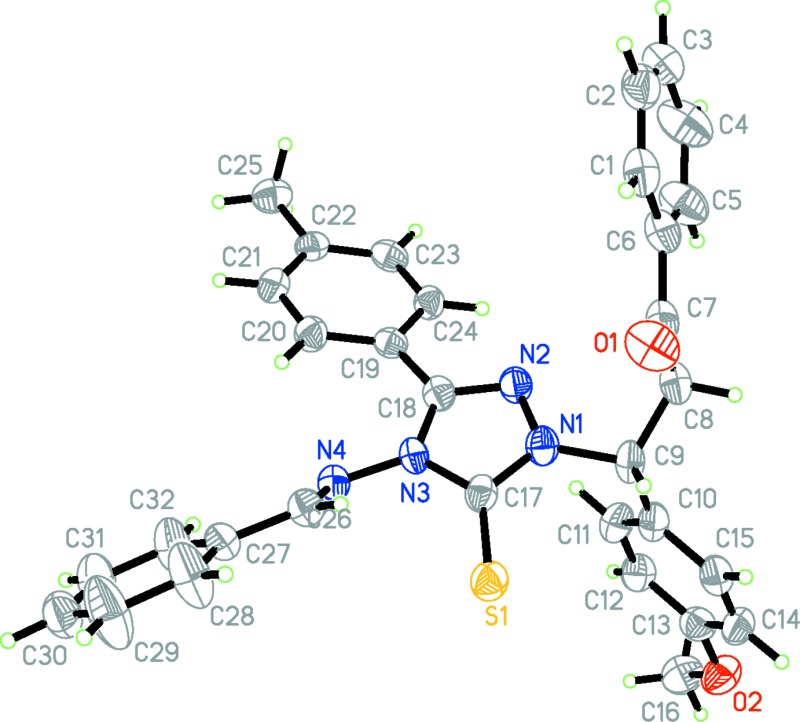
View of the title molecule showing the atom-labelling scheme. Displacement ellipsoids are drawn at the 30% probability level.

. DOI: 10.1107/S2056989015023804/wm5250fig1.tif
View of the title mol­ecule showing the atom-labelling scheme. Displacement ellipsoids are drawn at the 30% probability level.

CCDC reference: 1441814

Additional supporting information:  crystallographic information; 3D view; checkCIF report

## Figures and Tables

**Table 1:** Hydrogen-bond geometry (Å, °)

*D*—H⋯*A*	*D*—H	H⋯*A*	*D*⋯*A*	*D*—H⋯*A*
C11—H11⋯N2	0.95	2.60	3.250 (4)	126
C20—H20⋯N4	0.95	2.39	2.990 (4)	121
C24—H24⋯O1^i^	0.95	2.58	3.464 (4)	156
C26—H26⋯S1	0.95	2.71	3.188 (3)	112

Symmetry code: (i) 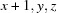.

## supplementary crystallographic information

### S1. Experimental

The title compound was synthesized by the reaction of benzaldehyde (2.0 mmol) and 3-(4-amino-5-thioxo-3-(*p*-tolyl)-4,5-dihydro-1*H*-1,2,4-triazol-1-yl)-3-(4-methoxyphenyl)-1-phenylpropan-1-one (2.0 mmol) under reflux in ethanol. The reaction progress was monitored *via* TLC. The resulting precipitate was filtered off, washed with cold ethanol, dried and purified to give the target product as a colorless solid in 75% yield. Crystals of the title compound suitable for single-crystal X-ray analysis were grown by slow evaporation of a chloroform-ethanol (1:1, *v*/*v*) mixture.

### S2. Refinement

All H atoms were positioned geometrically and refined as riding (C—H = 0.95–0.99 Å) and allowed to ride on their parent atoms, with *U*_iso_(H) = 1.2*U*_eq_(C)_aromatic_ or 1.5*U*_eq_(*C*)_methyl_.

### Crystal data

**Table:**

C_32_H_28_N_4_O_2_S	*Z* = 2
*M**_r_* = 532.64	*F*(000) = 560
Triclinic, *P*1	*D*_x_ = 1.302 Mg m^−^^3^
Hall symbol: -P 1	Mo *K*α radiation, λ = 0.71073 Å
*a* = 6.2343 (6) Å	Cell parameters from 4230 reflections
*b* = 14.6968 (14) Å	θ = 1.4–27.9°
*c* = 15.0540 (17) Å	µ = 0.16 mm^−^^1^
α = 90.234 (9)°	*T* = 113 K
β = 92.612 (13)°	Prism, colorless
γ = 99.497 (10)°	0.20 × 0.18 × 0.12 mm
*V* = 1358.9 (2) Å^3^	

### Data collection

**Table:**

Rigaku Saturn CCD area-detector diffractometer	4778 independent reflections
Radiation source: rotating anode	3450 reflections with *I* > 2σ(*I*)
Multilayer monochromator	*R*_int_ = 0.057
Detector resolution: 14.22 pixels mm^-1^	θ_max_ = 25.0°, θ_min_ = 1.4°
φ and ω scans	*h* = −7→7
Absorption correction: multi-scan (*CrystalClear*; Rigaku/MSC, 2005)	*k* = −17→17
*T*_min_ = 0.969, *T*_max_ = 0.982	*l* = −15→17
11718 measured reflections	

### Refinement

**Table:**

Refinement on *F*^2^	Primary atom site location: structure-invariant direct methods
Least-squares matrix: full	Secondary atom site location: difference Fourier map
*R*[*F*^2^ > 2σ(*F*^2^)] = 0.065	Hydrogen site location: inferred from neighbouring sites
*wR*(*F*^2^) = 0.126	H-atom parameters constrained
*S* = 1.08	*w* = 1/[σ^2^(*F*_o_^2^) + (0.0362*P*)^2^] where *P* = (*F*_o_^2^ + 2*F*_c_^2^)/3
4778 reflections	(Δ/σ)_max_ < 0.001
354 parameters	Δρ_max_ = 0.23 e Å^−^^3^
0 restraints	Δρ_min_ = −0.25 e Å^−^^3^

### Special details

**Table:**

Geometry. All e.s.d.'s (except the e.s.d. in the dihedral angle between two l.s. planes) are estimated using the full covariance matrix. The cell e.s.d.'s are taken into account individually in the estimation of e.s.d.'s in distances, angles and torsion angles; correlations between e.s.d.'s in cell parameters are only used when they are defined by crystal symmetry. An approximate (isotropic) treatment of cell e.s.d.'s is used for estimating e.s.d.'s involving l.s. planes.
Refinement. Refinement of *F*^2^ against ALL reflections. The weighted *R*-factor *wR* and goodness of fit *S* are based on *F*^2^, conventional *R*-factors *R* are based on *F*, with *F* set to zero for negative *F*^2^. The threshold expression of *F*^2^ > σ(*F*^2^) is used only for calculating *R*-factors(gt) *etc*. and is not relevant to the choice of reflections for refinement. *R*-factors based on *F*^2^ are statistically about twice as large as those based on *F*, and *R*- factors based on ALL data will be even larger.

### Fractional atomic coordinates and isotropic or equivalent isotropic displacement parameters (Å^2^)

**Table:**

	*x*	*y*	*z*	*U*_iso_*/*U*_eq_	
S1	0.30821 (12)	0.76134 (6)	0.33347 (5)	0.0326 (2)	
O1	0.2917 (3)	0.84691 (17)	0.03956 (14)	0.0466 (6)	
O2	1.1239 (3)	1.11680 (15)	0.44187 (14)	0.0418 (6)	
N1	0.5946 (4)	0.78848 (17)	0.20148 (16)	0.0288 (6)	
N2	0.7452 (4)	0.74728 (17)	0.15811 (15)	0.0289 (6)	
N3	0.5736 (4)	0.65289 (16)	0.25500 (15)	0.0251 (6)	
N4	0.5344 (4)	0.57402 (17)	0.30854 (15)	0.0282 (6)	
C1	0.4101 (5)	0.7859 (2)	−0.1241 (2)	0.0378 (8)	
H1	0.2646	0.7674	−0.1071	0.045*	
C2	0.4689 (5)	0.7603 (2)	−0.2071 (2)	0.0414 (9)	
H2	0.3652	0.7234	−0.2461	0.050*	
C3	0.6790 (6)	0.7886 (2)	−0.2330 (2)	0.0463 (9)	
H3	0.7210	0.7713	−0.2897	0.056*	
C4	0.8290 (6)	0.8429 (3)	−0.1749 (2)	0.0573 (11)	
H4	0.9728	0.8639	−0.1928	0.069*	
C5	0.7699 (5)	0.8662 (3)	−0.0917 (2)	0.0477 (10)	
H5	0.8745	0.9019	−0.0522	0.057*	
C6	0.5594 (5)	0.8382 (2)	−0.0651 (2)	0.0341 (8)	
C7	0.4844 (5)	0.8637 (2)	0.0235 (2)	0.0345 (8)	
C8	0.6475 (5)	0.9123 (2)	0.09322 (19)	0.0337 (8)	
H8A	0.7925	0.8961	0.0831	0.040*	
H8B	0.6581	0.9798	0.0863	0.040*	
C9	0.5869 (5)	0.8870 (2)	0.1874 (2)	0.0318 (8)	
H9	0.4343	0.8974	0.1949	0.038*	
C10	0.7354 (5)	0.9461 (2)	0.2563 (2)	0.0305 (7)	
C11	0.9305 (5)	0.9220 (2)	0.2886 (2)	0.0354 (8)	
H11	0.9716	0.8665	0.2680	0.042*	
C12	1.0670 (5)	0.9768 (2)	0.3501 (2)	0.0336 (8)	
H12	1.1995	0.9589	0.3715	0.040*	
C13	1.0064 (5)	1.0585 (2)	0.3798 (2)	0.0330 (8)	
C14	0.8140 (5)	1.0844 (2)	0.3471 (2)	0.0346 (8)	
H14	0.7750	1.1409	0.3664	0.042*	
C15	0.6780 (5)	1.0282 (2)	0.2863 (2)	0.0333 (8)	
H15	0.5452	1.0459	0.2651	0.040*	
C16	1.3209 (5)	1.0916 (2)	0.4790 (2)	0.0444 (9)	
H16A	1.2897	1.0296	0.5039	0.067*	
H16B	1.3821	1.1357	0.5262	0.067*	
H16C	1.4259	1.0923	0.4324	0.067*	
C17	0.4885 (4)	0.7336 (2)	0.26328 (18)	0.0268 (7)	
C18	0.7328 (4)	0.6653 (2)	0.19328 (18)	0.0265 (7)	
C19	0.8735 (4)	0.5993 (2)	0.16637 (18)	0.0249 (7)	
C20	0.8278 (5)	0.5049 (2)	0.18174 (19)	0.0303 (7)	
H20	0.7020	0.4797	0.2123	0.036*	
C21	0.9675 (5)	0.4476 (2)	0.15206 (19)	0.0322 (8)	
H21	0.9358	0.3834	0.1637	0.039*	
C22	1.1507 (5)	0.4811 (2)	0.10621 (18)	0.0286 (7)	
C23	1.1946 (5)	0.5765 (2)	0.09149 (18)	0.0305 (7)	
H23	1.3200	0.6015	0.0606	0.037*	
C24	1.0587 (4)	0.6352 (2)	0.12106 (18)	0.0277 (7)	
H24	1.0917	0.6996	0.1105	0.033*	
C25	1.2951 (5)	0.4167 (2)	0.0723 (2)	0.0392 (8)	
H25A	1.2810	0.4134	0.0073	0.059*	
H25B	1.2510	0.3550	0.0966	0.059*	
H25C	1.4469	0.4399	0.0912	0.059*	
C26	0.3327 (5)	0.5466 (2)	0.32321 (19)	0.0315 (8)	
H26	0.2244	0.5776	0.2966	0.038*	
C27	0.2695 (5)	0.4680 (2)	0.38059 (19)	0.0321 (8)	
C28	0.0539 (5)	0.4429 (3)	0.4000 (2)	0.0554 (11)	
H28	−0.0527	0.4753	0.3742	0.066*	
C29	−0.0081 (6)	0.3699 (3)	0.4574 (3)	0.0706 (14)	
H29	−0.1573	0.3528	0.4699	0.085*	
C30	0.1416 (6)	0.3229 (2)	0.4957 (2)	0.0433 (9)	
H30	0.0983	0.2742	0.5357	0.052*	
C31	0.3562 (6)	0.3468 (2)	0.4759 (2)	0.0470 (9)	
H31	0.4614	0.3132	0.5011	0.056*	
C32	0.4202 (5)	0.4186 (2)	0.4199 (2)	0.0468 (10)	
H32	0.5698	0.4350	0.4079	0.056*	

### Atomic displacement parameters (Å^2^)

**Table:**

	*U*^11^	*U*^22^	*U*^33^	*U*^12^	*U*^13^	*U*^23^
S1	0.0324 (5)	0.0306 (5)	0.0362 (5)	0.0091 (4)	0.0037 (4)	0.0018 (4)
O1	0.0281 (13)	0.0631 (18)	0.0462 (15)	0.0003 (13)	0.0005 (11)	0.0048 (12)
O2	0.0445 (14)	0.0375 (15)	0.0433 (14)	0.0086 (12)	−0.0049 (11)	−0.0091 (11)
N1	0.0253 (14)	0.0267 (15)	0.0349 (16)	0.0053 (12)	0.0010 (12)	0.0043 (12)
N2	0.0302 (15)	0.0265 (15)	0.0305 (15)	0.0058 (13)	0.0030 (11)	0.0035 (11)
N3	0.0264 (14)	0.0225 (14)	0.0266 (14)	0.0046 (12)	−0.0004 (11)	0.0059 (11)
N4	0.0312 (15)	0.0244 (15)	0.0294 (15)	0.0048 (12)	0.0031 (12)	0.0024 (11)
C1	0.0347 (19)	0.031 (2)	0.046 (2)	0.0024 (16)	−0.0027 (16)	0.0098 (16)
C2	0.047 (2)	0.032 (2)	0.042 (2)	0.0002 (18)	−0.0043 (17)	0.0038 (16)
C3	0.059 (3)	0.044 (2)	0.036 (2)	0.010 (2)	0.0048 (19)	0.0079 (17)
C4	0.046 (2)	0.081 (3)	0.042 (2)	0.003 (2)	0.0046 (19)	0.013 (2)
C5	0.041 (2)	0.061 (3)	0.037 (2)	−0.002 (2)	−0.0027 (17)	0.0081 (18)
C6	0.0324 (19)	0.034 (2)	0.035 (2)	0.0054 (16)	−0.0014 (15)	0.0093 (15)
C7	0.038 (2)	0.0267 (19)	0.038 (2)	0.0054 (16)	−0.0018 (16)	0.0073 (14)
C8	0.0357 (19)	0.0268 (19)	0.038 (2)	0.0027 (16)	0.0004 (15)	0.0086 (14)
C9	0.0334 (18)	0.0216 (18)	0.041 (2)	0.0068 (15)	0.0029 (15)	0.0067 (14)
C10	0.0311 (18)	0.0236 (18)	0.0374 (19)	0.0054 (15)	0.0040 (15)	0.0044 (14)
C11	0.0353 (19)	0.029 (2)	0.044 (2)	0.0100 (16)	0.0020 (16)	−0.0002 (15)
C12	0.0306 (18)	0.033 (2)	0.0375 (19)	0.0063 (16)	0.0006 (15)	0.0019 (15)
C13	0.0341 (19)	0.034 (2)	0.0299 (19)	0.0018 (16)	0.0037 (15)	0.0012 (14)
C14	0.040 (2)	0.0265 (19)	0.040 (2)	0.0107 (16)	0.0069 (16)	0.0007 (15)
C15	0.0337 (19)	0.030 (2)	0.037 (2)	0.0057 (16)	0.0040 (15)	0.0073 (15)
C16	0.041 (2)	0.045 (2)	0.045 (2)	0.0032 (18)	−0.0027 (17)	−0.0065 (17)
C17	0.0267 (17)	0.0237 (18)	0.0295 (18)	0.0043 (14)	−0.0040 (13)	0.0009 (13)
C18	0.0250 (17)	0.0257 (18)	0.0273 (18)	0.0008 (14)	−0.0021 (14)	0.0017 (13)
C19	0.0232 (16)	0.0288 (18)	0.0219 (16)	0.0029 (14)	−0.0010 (13)	−0.0001 (13)
C20	0.0294 (17)	0.0278 (19)	0.0330 (18)	0.0019 (15)	0.0049 (14)	0.0013 (14)
C21	0.0371 (19)	0.0246 (18)	0.0336 (19)	0.0010 (16)	0.0028 (15)	−0.0020 (14)
C22	0.0309 (18)	0.0310 (19)	0.0236 (17)	0.0052 (15)	−0.0012 (14)	−0.0036 (13)
C23	0.0252 (17)	0.040 (2)	0.0256 (17)	0.0026 (16)	0.0028 (13)	0.0024 (14)
C24	0.0287 (17)	0.0239 (18)	0.0286 (18)	0.0001 (15)	−0.0015 (14)	0.0009 (13)
C25	0.0389 (19)	0.044 (2)	0.036 (2)	0.0107 (18)	0.0033 (15)	−0.0045 (16)
C26	0.0346 (19)	0.030 (2)	0.0298 (18)	0.0058 (16)	0.0009 (15)	0.0017 (14)
C27	0.0367 (19)	0.0299 (19)	0.0289 (18)	0.0024 (16)	0.0044 (14)	0.0033 (14)
C28	0.034 (2)	0.060 (3)	0.075 (3)	0.011 (2)	0.0095 (19)	0.036 (2)
C29	0.042 (2)	0.073 (3)	0.099 (4)	0.007 (2)	0.022 (2)	0.047 (3)
C30	0.050 (2)	0.038 (2)	0.042 (2)	0.0069 (19)	0.0027 (17)	0.0100 (16)
C31	0.047 (2)	0.040 (2)	0.054 (2)	0.0081 (19)	−0.0018 (18)	0.0150 (18)
C32	0.037 (2)	0.041 (2)	0.062 (3)	0.0067 (18)	0.0026 (18)	0.0205 (19)

### Geometric parameters (Å, º)

**Table:**

S1—C17	1.674 (3)	C13—C14	1.388 (4)
O1—C7	1.221 (3)	C14—C15	1.390 (4)
O2—C13	1.366 (4)	C14—H14	0.9500
O2—C16	1.431 (3)	C15—H15	0.9500
N1—C17	1.356 (3)	C16—H16A	0.9800
N1—N2	1.384 (3)	C16—H16B	0.9800
N1—C9	1.472 (3)	C16—H16C	0.9800
N2—C18	1.310 (3)	C18—C19	1.478 (4)
N3—C18	1.381 (3)	C19—C20	1.392 (4)
N3—C17	1.385 (3)	C19—C24	1.397 (4)
N3—N4	1.409 (3)	C20—C21	1.393 (4)
N4—C26	1.285 (3)	C20—H20	0.9500
C1—C2	1.387 (4)	C21—C22	1.383 (4)
C1—C6	1.388 (4)	C21—H21	0.9500
C1—H1	0.9500	C22—C23	1.404 (4)
C2—C3	1.381 (4)	C22—C25	1.512 (4)
C2—H2	0.9500	C23—C24	1.390 (4)
C3—C4	1.396 (5)	C23—H23	0.9500
C3—H3	0.9500	C24—H24	0.9500
C4—C5	1.379 (4)	C25—H25A	0.9800
C4—H4	0.9500	C25—H25B	0.9800
C5—C6	1.387 (4)	C25—H25C	0.9800
C5—H5	0.9500	C26—C27	1.458 (4)
C6—C7	1.501 (4)	C26—H26	0.9500
C7—C8	1.517 (4)	C27—C28	1.378 (4)
C8—C9	1.516 (4)	C27—C32	1.392 (4)
C8—H8A	0.9900	C28—C29	1.396 (4)
C8—H8B	0.9900	C28—H28	0.9500
C9—C10	1.525 (4)	C29—C30	1.359 (4)
C9—H9	1.0000	C29—H29	0.9500
C10—C11	1.389 (4)	C30—C31	1.373 (4)
C10—C15	1.394 (4)	C30—H30	0.9500
C11—C12	1.387 (4)	C31—C32	1.372 (4)
C11—H11	0.9500	C31—H31	0.9500
C12—C13	1.395 (4)	C32—H32	0.9500
C12—H12	0.9500		
			
C13—O2—C16	117.5 (2)	C10—C15—H15	119.8
C17—N1—N2	113.2 (2)	O2—C16—H16A	109.5
C17—N1—C9	126.6 (2)	O2—C16—H16B	109.5
N2—N1—C9	119.4 (2)	H16A—C16—H16B	109.5
C18—N2—N1	104.8 (2)	O2—C16—H16C	109.5
C18—N3—C17	109.2 (2)	H16A—C16—H16C	109.5
C18—N3—N4	122.9 (2)	H16B—C16—H16C	109.5
C17—N3—N4	127.3 (2)	N1—C17—N3	102.6 (2)
C26—N4—N3	114.4 (2)	N1—C17—S1	127.3 (2)
C2—C1—C6	121.4 (3)	N3—C17—S1	130.1 (2)
C2—C1—H1	119.3	N2—C18—N3	110.0 (2)
C6—C1—H1	119.3	N2—C18—C19	122.0 (3)
C3—C2—C1	119.7 (3)	N3—C18—C19	127.9 (3)
C3—C2—H2	120.2	C20—C19—C24	119.4 (3)
C1—C2—H2	120.2	C20—C19—C18	123.7 (3)
C2—C3—C4	119.3 (3)	C24—C19—C18	116.8 (3)
C2—C3—H3	120.3	C19—C20—C21	119.7 (3)
C4—C3—H3	120.3	C19—C20—H20	120.2
C5—C4—C3	120.4 (3)	C21—C20—H20	120.2
C5—C4—H4	119.8	C22—C21—C20	122.2 (3)
C3—C4—H4	119.8	C22—C21—H21	118.9
C4—C5—C6	120.7 (3)	C20—C21—H21	118.9
C4—C5—H5	119.6	C21—C22—C23	117.4 (3)
C6—C5—H5	119.6	C21—C22—C25	120.9 (3)
C5—C6—C1	118.4 (3)	C23—C22—C25	121.7 (3)
C5—C6—C7	123.0 (3)	C24—C23—C22	121.5 (3)
C1—C6—C7	118.5 (3)	C24—C23—H23	119.2
O1—C7—C6	120.3 (3)	C22—C23—H23	119.2
O1—C7—C8	119.6 (3)	C23—C24—C19	119.8 (3)
C6—C7—C8	120.0 (3)	C23—C24—H24	120.1
C9—C8—C7	112.9 (3)	C19—C24—H24	120.1
C9—C8—H8A	109.0	C22—C25—H25A	109.5
C7—C8—H8A	109.0	C22—C25—H25B	109.5
C9—C8—H8B	109.0	H25A—C25—H25B	109.5
C7—C8—H8B	109.0	C22—C25—H25C	109.5
H8A—C8—H8B	107.8	H25A—C25—H25C	109.5
N1—C9—C8	109.4 (2)	H25B—C25—H25C	109.5
N1—C9—C10	110.5 (2)	N4—C26—C27	120.2 (3)
C8—C9—C10	111.9 (3)	N4—C26—H26	119.9
N1—C9—H9	108.3	C27—C26—H26	119.9
C8—C9—H9	108.3	C28—C27—C32	118.0 (3)
C10—C9—H9	108.3	C28—C27—C26	119.5 (3)
C11—C10—C15	118.5 (3)	C32—C27—C26	122.5 (3)
C11—C10—C9	122.0 (3)	C27—C28—C29	120.2 (3)
C15—C10—C9	119.4 (3)	C27—C28—H28	119.9
C12—C11—C10	121.9 (3)	C29—C28—H28	119.9
C12—C11—H11	119.1	C30—C29—C28	121.0 (3)
C10—C11—H11	119.1	C30—C29—H29	119.5
C11—C12—C13	118.8 (3)	C28—C29—H29	119.5
C11—C12—H12	120.6	C29—C30—C31	119.1 (3)
C13—C12—H12	120.6	C29—C30—H30	120.4
O2—C13—C14	115.6 (3)	C31—C30—H30	120.4
O2—C13—C12	124.3 (3)	C32—C31—C30	120.6 (3)
C14—C13—C12	120.1 (3)	C32—C31—H31	119.7
C13—C14—C15	120.3 (3)	C30—C31—H31	119.7
C13—C14—H14	119.9	C31—C32—C27	121.1 (3)
C15—C14—H14	119.9	C31—C32—H32	119.5
C14—C15—C10	120.4 (3)	C27—C32—H32	119.5
C14—C15—H15	119.8		
			
C17—N1—N2—C18	0.1 (3)	N2—N1—C17—N3	−2.2 (3)
C9—N1—N2—C18	171.3 (2)	C9—N1—C17—N3	−172.6 (3)
C18—N3—N4—C26	−143.4 (3)	N2—N1—C17—S1	175.6 (2)
C17—N3—N4—C26	46.8 (4)	C9—N1—C17—S1	5.2 (4)
C6—C1—C2—C3	−1.3 (5)	C18—N3—C17—N1	3.4 (3)
C1—C2—C3—C4	−0.1 (5)	N4—N3—C17—N1	174.3 (2)
C2—C3—C4—C5	1.6 (5)	C18—N3—C17—S1	−174.3 (2)
C3—C4—C5—C6	−1.6 (6)	N4—N3—C17—S1	−3.4 (4)
C4—C5—C6—C1	0.2 (5)	N1—N2—C18—N3	2.1 (3)
C4—C5—C6—C7	−178.3 (3)	N1—N2—C18—C19	−178.4 (2)
C2—C1—C6—C5	1.2 (5)	C17—N3—C18—N2	−3.6 (3)
C2—C1—C6—C7	179.8 (3)	N4—N3—C18—N2	−175.1 (2)
C5—C6—C7—O1	171.1 (3)	C17—N3—C18—C19	177.0 (3)
C1—C6—C7—O1	−7.4 (5)	N4—N3—C18—C19	5.6 (4)
C5—C6—C7—C8	−7.7 (5)	N2—C18—C19—C20	−161.1 (3)
C1—C6—C7—C8	173.8 (3)	N3—C18—C19—C20	18.2 (4)
O1—C7—C8—C9	33.4 (4)	N2—C18—C19—C24	17.2 (4)
C6—C7—C8—C9	−147.8 (3)	N3—C18—C19—C24	−163.5 (3)
C17—N1—C9—C8	−157.2 (3)	C24—C19—C20—C21	0.2 (4)
N2—N1—C9—C8	32.8 (3)	C18—C19—C20—C21	178.5 (3)
C17—N1—C9—C10	79.1 (4)	C19—C20—C21—C22	−0.9 (4)
N2—N1—C9—C10	−90.8 (3)	C20—C21—C22—C23	1.0 (4)
C7—C8—C9—N1	65.0 (3)	C20—C21—C22—C25	−178.0 (3)
C7—C8—C9—C10	−172.2 (2)	C21—C22—C23—C24	−0.5 (4)
N1—C9—C10—C11	34.7 (4)	C25—C22—C23—C24	178.6 (3)
C8—C9—C10—C11	−87.6 (3)	C22—C23—C24—C19	−0.1 (4)
N1—C9—C10—C15	−147.2 (3)	C20—C19—C24—C23	0.3 (4)
C8—C9—C10—C15	90.6 (3)	C18—C19—C24—C23	−178.1 (2)
C15—C10—C11—C12	0.7 (5)	N3—N4—C26—C27	−177.5 (3)
C9—C10—C11—C12	178.8 (3)	N4—C26—C27—C28	176.1 (3)
C10—C11—C12—C13	−0.3 (5)	N4—C26—C27—C32	−1.3 (5)
C16—O2—C13—C14	178.5 (3)	C32—C27—C28—C29	−0.1 (6)
C16—O2—C13—C12	−0.5 (5)	C26—C27—C28—C29	−177.7 (4)
C11—C12—C13—O2	178.2 (3)	C27—C28—C29—C30	0.6 (7)
C11—C12—C13—C14	−0.9 (5)	C28—C29—C30—C31	−1.4 (6)
O2—C13—C14—C15	−177.5 (3)	C29—C30—C31—C32	1.7 (6)
C12—C13—C14—C15	1.6 (5)	C30—C31—C32—C27	−1.2 (6)
C13—C14—C15—C10	−1.2 (5)	C28—C27—C32—C31	0.4 (5)
C11—C10—C15—C14	0.0 (5)	C26—C27—C32—C31	177.9 (3)
C9—C10—C15—C14	−178.2 (3)		

### Hydrogen-bond geometry (Å, º)

**Table:**

*D*—H···*A*	*D*—H	H···*A*	*D*···*A*	*D*—H···*A*
C11—H11···N2	0.95	2.60	3.250 (4)	126
C20—H20···N4	0.95	2.39	2.990 (4)	121
C24—H24···O1^i^	0.95	2.58	3.464 (4)	156
C26—H26···S1	0.95	2.71	3.188 (3)	112

Symmetry code: (i) *x*+1, *y*, *z*.
