# Supplementary figures and images for: Crystal structure of (E)-3-[4-(benzyl­idene­amino)-5-sulfanyl­idene-3-(p-tol­yl)-4,5-di­hydro-1H-1,2,4-triazol-1-yl]-3-(4-meth­oxy­phen­yl)-1-phenyl­propan-1-one
Source: Acta Crystallogr E Crystallogr Commun. 2015 Dec 24;71(Pt 12):o1080–1. doi: 10.1107/S2056989015023804 (PMC4719989; doi:10.1107/S2056989015023804)

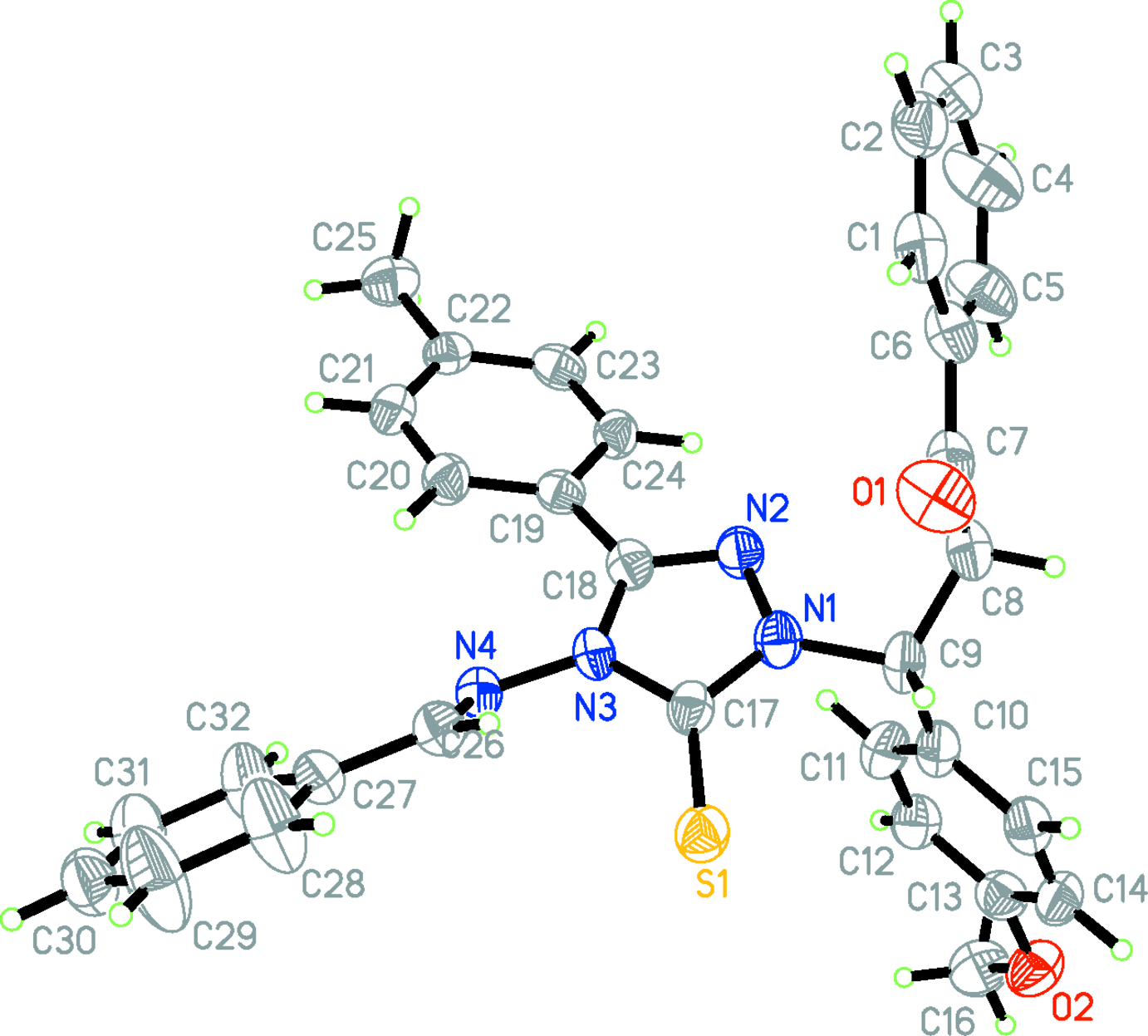

Supplement: Supplementary file 4 [file e-71-o1080-fig1.tif]
